# Reproduction study using public data of: Development and validation of a deep learning algorithm for detection of diabetic retinopathy in retinal fundus photographs

**DOI:** 10.1371/journal.pone.0217541

**Published:** 2019-06-06

**Authors:** Mike Voets, Kajsa Møllersen, Lars Ailo Bongo

**Affiliations:** 1 Department of Computer Science, UiT The Arctic University of Norway, Tromsø, Norway; 2 Department of Community Medicine, UiT The Arctic University of Norway, Tromsø, Norway; Liverpool John Moores University, UNITED KINGDOM

## Abstract

We have attempted to reproduce the results in *Development and validation of a deep learning algorithm for detection of diabetic retinopathy in retinal fundus photographs*, published in JAMA 2016; 316(22), using publicly available data sets. We re-implemented the main method in the original study since the source code is not available. The original study used non-public fundus images from EyePACS and three hospitals in India for training. We used a different EyePACS data set from Kaggle. The original study used the benchmark data set Messidor-2 to evaluate the algorithm’s performance. We used another distribution of the Messidor-2 data set, since the original data set is no longer available. In the original study, ophthalmologists re-graded all images for diabetic retinopathy, macular edema, and image gradability. We have one diabetic retinopathy grade per image for our data sets, and we assessed image gradability ourselves. We were not able to reproduce the original study’s results with publicly available data. Our algorithm’s area under the receiver operating characteristic curve (AUC) of 0.951 (95% CI, 0.947-0.956) on the Kaggle EyePACS test set and 0.853 (95% CI, 0.835-0.871) on Messidor-2 did not come close to the reported AUC of 0.99 on both test sets in the original study. This may be caused by the use of a single grade per image, or different data. This study shows the challenges of reproducing deep learning method results, and the need for more replication and reproduction studies to validate deep learning methods, especially for medical image analysis. Our source code and instructions are available at: https://github.com/mikevoets/jama16-retina-replication.

## Introduction

Being able to replicate a scientific paper by strictly following the described methods is a cornerstone of science. Replicability is essential for the development of medical technologies based on published results. However, there is an emerging concern that many studies are not replicable [1], including for bio-medical research [2], computational sciences [3, 4], and recently for machine learning [5].

Deep learning has become a hot topic within machine learning due to its promising performance of finding patterns in large data sets. There are dozens of libraries that make deep learning methods easily available for any developer. This has consequently led to an increase of published articles that demonstrate the feasibility of applying deep learning in practice, particularly for image classification [6, 7]. However, there is an emerging need to show that studies are replicable, and hence be used to develop new medical analysis solutions. Ideally, the data set and the source code are published, so that other researchers can verify the results by using the same or other data. However, this is not always practical, for example for sensitive data, or for methods with commercial value [4, 5].

If a replication study can not be conducted due to restricted access to the data, a reproduction study can be performed on a similar data set. Deviations in the results can be due to either differences in the data, or insufficient description of the method, and more details in the method description can reveal the source of deviation.

In this study, we make an attempt to reproduce the results of a well known deep learning algorithm: *Development and validation of a deep learning algorithm for detection of diabetic retinopathy in retinal fundus photographs*, published in JAMA 2016; 316(22) [8]. As of March 2019, this article had been cited 906 times [9]. We chose to reproduce the results of this study because it is a well-known and high-impact study within the medical field, the source code has not been published, and to our knowledge the algorithm has not been reproduced.

The main findings of the original study have been verified in other publications for detection of diabetic retinopathy [10–12] with high performance. One such method, IDx-DR, was FDA approved in 2018 [13]. We cannot directly compare our results with these algorithms, since they use different data sets for evaluation of performance. A list of the result of our algorithm and many other studies is available in [14]. These studies confirm the main findings of [8]; that deep learning can be used to automatically detect diabetic retinopathy. We assess [8] to be most promising regarding reproducibility, due to its detailed method descriptions.

The original study describes an algorithm (hereby referred to as the original algorithm) for detection of referable diabetic retinopathy (rDR) in retinal fundus photographs. The algorithm is trained and validated using 118 419 fundus images retrieved from EyePACS and from three eye hospitals in India. The original algorithm’s performance was evaluated on 2 test sets, and achieved an area under the receiver operating characteristic curve (AUC) for detecting rDR of 0.99 for both the EyePACS-1 and the Messidor-2 test sets. Two operating points were selected for high sensitivity and specificity. The operating point for high specificity had 90.3% and 87.0% sensitivity and 98.1% and 98.5% specificity for the EyePACS-1 and Messidor-2 test sets, whereas the operating point for high sensitivity had 97.5% and 96.1% sensitivity and 93.4% and 93.9% specificity, respectively.

To re-implement the original algorithm for detection of rDR, we used similar images from a publicly available EyePACS data set on Kaggle for training and validation, and we used a subset from the EyePACS data set and images from the public Messidor-2 data set for performance evaluation. Our final hyper-parameter settings were different than in the original study, because the best choice may be different due to differences in our data and labels. Our objective is to compare the performance of the original rDR detection algorithm to the results of our reproduced algorithm, taking into account potential deviations in the data sets, having fewer grades, and differences in normalization methods and other hyper-parameter settings.

We were not able to reproduce the original study’s results with publicly available data. Our algorithm’s AUC for detecting rDR for our EyePACS and Messidor-2 test sets were 0.951 (95% CI, 0.947-0.956) and 0.853 (95% CI, 0.835-0.871), respectively. The operating point for high specificity had 83.6% and 68.7% sensitivity and 92.0% and 88.5% specificity for our EyePACS and Messidor-2 test sets, and the operating point for high sensitivity had 90.6% and 81.8% sensitivity and 84.7% and 71.2% specificity. The results can differ for four reasons. First, we used public retinal images from Kaggle with only one grade per image, whereas in the original study the non-public retinal images from multiple sources, and these were re-graded multiple times. Second, the hyper-parameters used in the original study may have been tuned better according to their data. Third, there might be errors in the original study or methodology. The last possible reason is that we may have done something wrong with reproducing the method by having misinterpreted the methodology. We cannot know for sure which of the four reasons has led to our considerably worse performance.

We do not believe our results invalidate the main findings of the original study. However, our result gives a general insight into the challenges of reproducing studies that do not use publicly available data and publish source code, and it motivates the need for additional replication and reproduction studies in deep learning. We have published our source code with instructions for how to use it with public data. This gives others the opportunity to improve the reproduced algorithm.

## Materials and methods

### Data sets

The data sets consist of images of the retinal fundus acquired for diabetic retinopathy screening. Any other information regarding the patient is not part of the data sets. Each image is graded according to severity of symptoms (see Section Grading).

The original study obtained 128 175 retinal fundus images from EyePACS in the US and from three eye hospitals in India. 118 419 macula-centered images from this data set were used for algorithm training and validation (referred to as *development set*, divided into *training* and *tuning set* in the original study). To evaluate the performance of the algorithm, the original study used two data sets (referred to as *validation sets* in the original study). For evaluating an algorithm’s performance, the term test set is commonly used. The first test set was a randomly sampled set of 9963 images retrieved at EyePACS screening sites between May 2015 and October 2015. The second test set was the publicly available Messidor-2 data set [15, 16], consisting of 1748 images. We provide an overview of the differences in image distribution used in our reproduction study compared with the original study in S1 Fig.

We obtained images for training, validation and testing from two sources: EyePACS from a Kaggle competition [17], and the Messidor-2 set that was used in the original study. The Messidor-2 set is a benchmark for algorithms that detect diabetic retinopathy. We randomly sampled the Kaggle EyePACS data set consisting of 88 702 images into a training and validation set of 57 146 images and a test set of 8790 images. The leftover images were mostly images graded as having no diabetic retinopathy and were not used for training the algorithm. The reason for the number of images in our training and validation set is to keep the same balance for the binary rDR class as in the original study’s training and validation set. Our EyePACS test set has an identical amount of images and balance for the binary rDR class as in the original study’s EyePACS test set. We used all 1748 images from the Messidor-2 test set.

### Grading

The images used for the algorithm training and testing in the original study were all graded by ophthalmologists for image quality (gradability), the presence of diabetic retinopathy, and macular edema. We did not have grades for macular edema for all our images, so we did not train our algorithm to detect macular edema.

**Fig 1:**
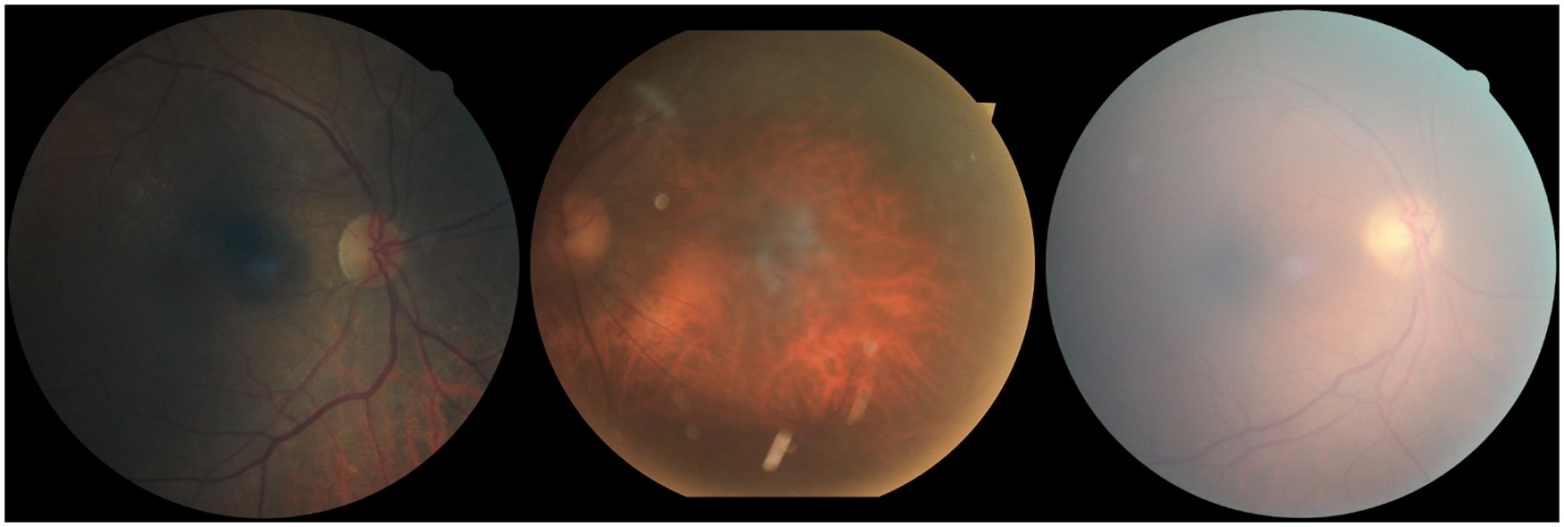
Ungradable images. Examples of ungradable images because they are either out of focus, under-, or overexposed.

**Fig 2:**
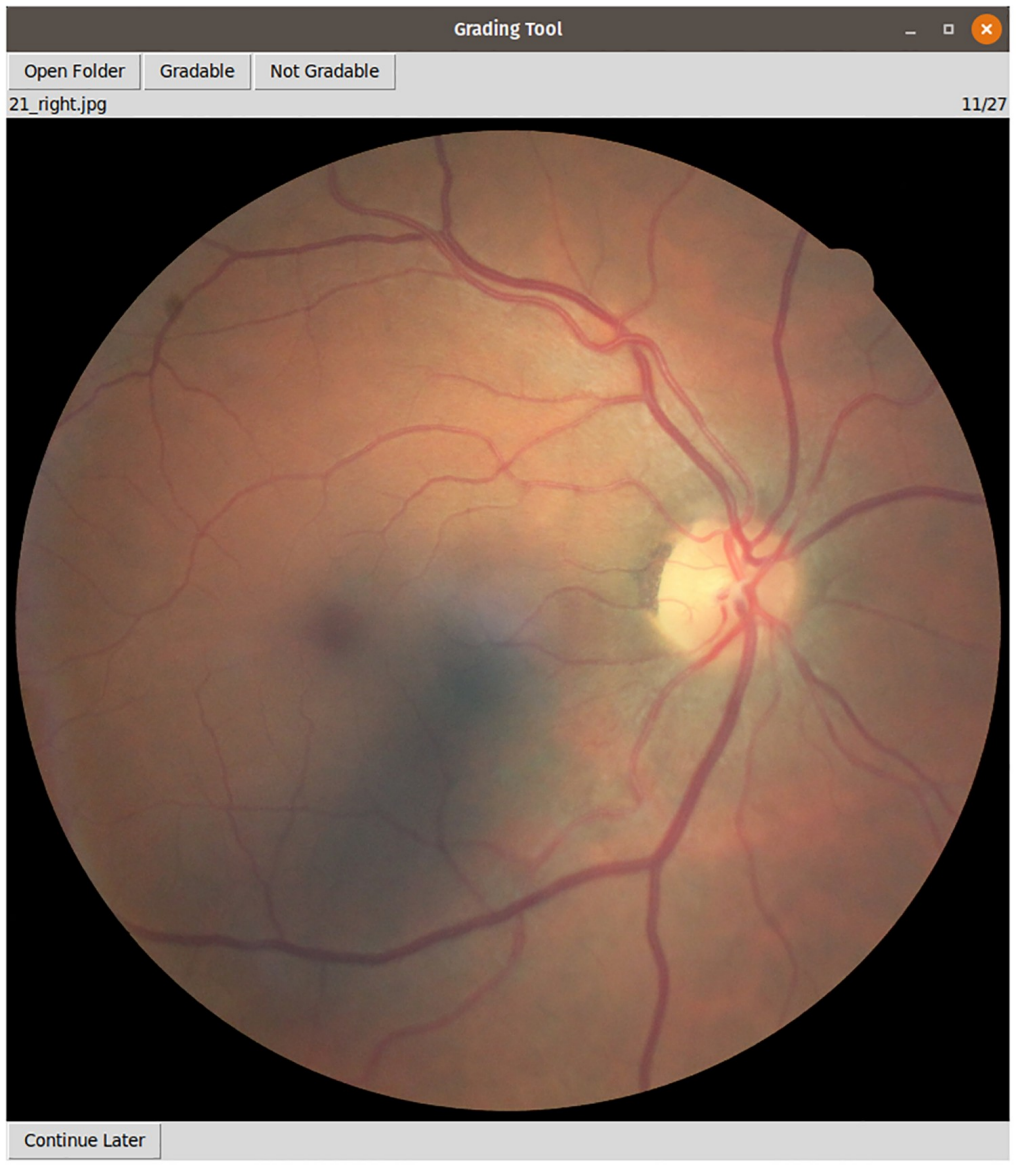
Grading tool. Screenshot of grading tool used to assess gradability for all images.

Kaggle [18] describes that some of the images in their EyePACS distribution may consist of noise, contain artifacts, be out of focus, or be over- or underexposed (Fig 1). [19] states further that 75% of the EyePACS images via Kaggle are estimated gradable. For this study one of the authors (MV) graded all Kaggle and Messidor-Original images on their image quality with a simple grading tool (Fig 2). MV is not a licensed ophthalmologist, but we assume fundus image quality can be reliably graded by non-experts. We used the “Grading Instructions” in the Supplement of the original study to assess image quality. We publish the image quality grades with the source code. Images of at least adequate quality were considered gradable.

In the original study, diabetic retinopathy was graded according to the International Clinical Diabetic Retinopathy scale [20], with no, mild, moderate, severe or proliferative severity.

The Kaggle EyePACS set was graded for the presence of diabetic retinopathy using the same international scaling standard as used in the original study. We have only one diagnosis grade for each image. Kaggle does not give more information about where the data is from. The Messidor-2 test set and its diabetic retinopathy grades were made available by Ambramoff [21]. The grades are per-patient, which is the maximum grade of two eyes Ambramoff [21]. The original study uses per-image grades. There is also an additional criteria for referable diabetic macular edema, presence of 1 or more Microaneurysms within 1 Disc diameter, which is not used in the original study.

### Algorithm training

The objective of this study is to reproduce the results of the original study using publicly available data. We try to reproduce the method by following the original study’s methodology as accurately as possible. As in the original study, our algorithm is created through deep learning, which involves a procedure of training a neural network to perform the task of classifying images. We trained the algorithm with the same neural network architecture as in the original study: the InceptionV3 model proposed by Szegedy et al [22]. This neural network consists of a range of convolutional layers that transforms pixel intensities to local features before converting them into global features.

The fundus images from both training and test sets were preprocessed as described by the original study’s protocol for preprocessing. In all images the center and radius of the each fundus were located and resized such that each image gets a height and width of 299 pixels, with the fundus center in the middle of the image. A later article reports a list of data augmentation and training hyper-parameters for the trained algorithm in the original study [23]. We applied the same data augmentation settings in our image preprocessing procedure.

The original study used distributed stochastic gradient descent proposed by Dean et al [24] as the optimization function for training the parameters (i.e. weights) of the neural network. We believe their neural network was trained in parallel. We did not conduct any distributed training for our reproduced neural network, but it should not influence the accuracy of our algorithm. According to the hyper-parameters published in [23], the optimization method that was used in the original study was RMSProp. Therefore, we used RMSProp as our optimization procedure. The hyper-parameter list specifies a learning rate of 0.001, so we used this same learning rate for our algorithm training. We furthermore applied the same weight decay of 4 * 10^−5^.

As in the original study, we used batch normalization layers [25] after each convolutional layer. Our weights were also pre-initialized using weights from the neural network trained to predict objects in the ImageNet data set [26].

The neural network in the original study was trained to output multiple binary predictions: 1) whether the image was graded moderate or worse diabetic retinopathy (i.e. moderate, severe, or proliferative grades); 2) severe or worse diabetic retinopathy; 3) referable diabetic macular edema; or 4) fully gradable. The term referable diabetic retinopathy was defined in the original study as an image associated with either or both category 1) and 3). For the training data obtained in this reproduction study, only grades for diabetic retinopathy were present. That means that our neural network outputs only one binary prediction: moderate or worse diabetic retinopathy (referable diabetic retinopathy).

In this study, we split the training and validation sets like in the original study: 80% was used for training and 20% was used for validating the neural network. It is estimated that 25% of the Kaggle EyePACS set consists of ungradable images [19]. Therefore, we also assessed image gradability for all Kaggle EyePACS images, and we trained an algorithm with only gradable images. In the original study, the performance of an algorithm trained with only gradable images was also summarized. We do not use the image quality grades as an input for algorithm training.

Optimal hyper-parameter settings depend on the training data, so we conducted experiments to find the hyper-parameter settings that worked well for training and validating the algorithm with our publicly available data. We normalized the images to a [–1, 1] range before training.

### Algorithm validation

We validate the algorithm by measuring the performance of the resulting neural network by the area under the receiver operating characteristic curve (AUC) on a validation set, as in the original study. We find the area by thresholding the network’s output predictions, which are continuous numbers ranging from 0 to 1. By moving the operating threshold on the predictions, we obtain different results for sensitivity and specificity. We then plot sensitivity against 1–specificity for 200 thresholds. Finally, the AUC of the validation set is calculated, and becomes an indicator for how well the neural network detects referable diabetic retinopathy. We used the de facto standard of 200 thresholds for plotting the AUC.

The original paper describes that the AUC value of the validation set was used for the early-stopping criterion [27]; training is terminated when a peak AUC on the validation set is reached. This prevents overfitting the neural network on the training set. In our validation procedure, we also use the AUC calculated from the validation set as an early stopping criterion. To determine if a peak AUC is reached, we compared the AUC values between different validation checkpoints. To avoid stopping at a local maximum of the validation AUC function, our network may continue to perform training up to n epochs (i.e. patience of n epochs). The original study’s authors described that they most regularly adjusted the learning rate during validation (private communication). Other hyper-parameter settings like momentum, optimizer choice, batch size, settings for data augmentation, were fixed fairly early in the algorithm’s development. The values for these hyper-parameters were also borrowed from ImageNet pre-training, or earlier experiments.

We used ensemble learning [28] by training 10 networks on the same data set, and using the final prediction computed by taking the mean of the predictions of the ensemble. This was also done in the original study.

In the original study, additional experiments were conducted to evaluate the performance of the resulting algorithm based on the training set, compared with performance based on subsets of images and grades from the training set. We did not reproduce these experiments for two reasons. First, we chose to focus on reproducing the main results of the original paper. That is, the results of an algorithm detecting referable diabetic retinopathy. Second, we cannot perform subsampling of grades, as we only have one grade per image.

## Results

As for the early-stopping criterion at a peak AUC, we found that a patience of 10 epochs worked well. Our chosen requirement for a new peak AUC was a value of AUC that is larger than the previous peak value, with a minimum difference of 0.01.

**Fig 3:**
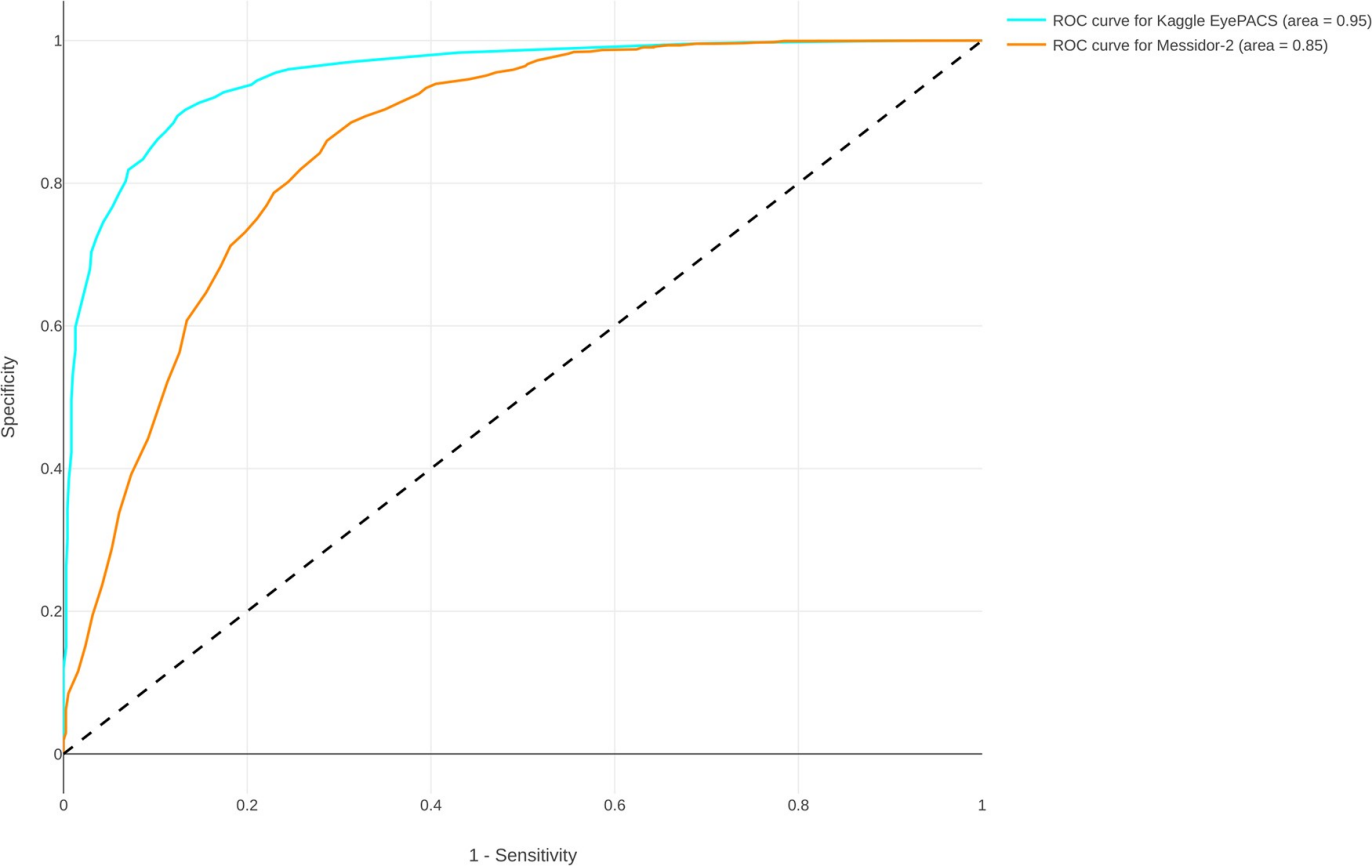
Reproduced results (AUC). Area under receiver operating characteristic curve for the reproduced algorithm.

The reproduced algorithm’s performance was evaluated on two independent test sets. We provide an overview of the differences in image distribution used in our reproduction study compared with the original study in S1 Fig. Our reproduced algorithm yielded an algorithm with an AUC of 0.951 (95% CI, 0.947-0.956) and 0.853 (95% CI, 0.835-0.871) on our Kaggle EyePACS test data set and Messidor-2, respectively (Fig 3 and Table 1). We observe that there is a large discrepancy between our AUC and the original study AUC. Lastly, we attempted training by excluding non-gradable images, but this did not increase algorithm performance.

**Table 1:** Reproduced results. Performance on test sets of reproduction, compared to results from the original study. The results of the original study are depicted in parenthesizes.

Reproduced results			
*Test set*	*High sensitivity*	*High specificity*	*AUC score*
Kaggle EyePACS test (orig. EyePACS-1)	90.6 (97.5)% sens.	83.6 (90.3)% sens.	0.951 (0.991)
	84.7 (93.4)% spec.	92.0 (98.1)% spec.	
Messidor-2	81.8 (96.1)% sens.	68.7 (87.0)% sens.	0.853 (0.990)
	71.2 (93.9)% spec.	88.5 (98.5)% spec.	

## Discussion

Our results show substantial performance differences between the original study’s algorithm and our reproduced algorithm. Even though we followed the methodology of the original study as closely as possible, our algorithm did not come close to the results in the original study. This is probably because our algorithms were trained with different public data, and because in the original study ophthalmologic experts re-graded all their images. According to the original study, the multiple grades per image should be used for ground truth, because the consensus will provide a more reliable measure of a model’s final predictive ability. This is not important for the training, but very important for the test set as shown in the original study results for experiments with only one grade per image, where their algorithm’s performance declines with 36%. This may explain the difference in performance (private communication with Dale Webster, Lily Peng, and Varun Gulshan). Another source for deviation between the original and the reproduced results is the quality of data and grading.

The original study’s authors suggested three additional possible sources for the difference between our EyePACS and Messidor-2 performance (private communication). First, and most significantly, training on our single Kaggle EyePACS data set may result in overfitting to the cameras and patient characteristics in that data set. Experiments performed by the original study’s team in the past have showed that this particular difference can have a large (10% AUC) impact on performance. Second, our per-patient grades for the Messidor-2 data set may result in overcalling of diabetic retinopathy. Finally, the original study model predicts additional outcomes that may slightly improve performance.

### Hyper-parameters

The main challenge in this reproduction study was to find optimal hyper-parameters for our data. The hyper-parameters were not published when we started this reproduction study. Later, hyper-parameters for training and data augmentation were published in [23], and then we retrained all algorithms with these hyper-parameters and data augmentation settings. To understand how we should further tune the hyper-parameters, we measured the Brier score on the training set and the AUC value on the validation set after each epoch of training. One possible reason for the algorithm having problems to converge may be the dimensions of the fundus images. As the original study suggests, the original fundus images were preprocessed and scaled down to a width and height of 299 pixels to be able to initialize the InceptionV3 network with ImageNet pre-trained weights, which have been trained with images of 299 by 299 pixels. We believe it is difficult for ophthalmologists to find lesions in fundus images of this size, so we assume the algorithm has difficulties with detecting lesions as well. [19] also points out this fact, and suggests re-training an entire network with larger fundus images and randomly initialized weights instead.

### Kaggle images

A potential drawback with the images from Kaggle is that it contains grades for diabetic retinopathy for all images. We found that 19.9% of these images is ungradable, and it is thus possible that the algorithm will “learn” features for ungradable images, and make predictions based on anomalies. This is likely to negatively contribute to the algorithm’s predictive performance, but we were not able to show a significant difference of performance between an algorithm trained on all images and an algorithm trained on only gradable images.

### Comparison to other studies

The review [14] compares our early results [29] with other deep learning models for diagnosis of diabetic retinopathy. The review shows that it is difficult to compare different methods because of three main reasons: 1) many methods are not tested on publicly available data; 2) there is no consensus on test metrics, e.g. specification of “high” sensitivity; 3) there is no consensus on how the classes should be defined, e.g. mild/moderate/severe. However, there is no doubt that the results from our first attempt of reproduction is lower than for studies that report original algorithms.

In the second attempt, after several of the hyper-parameters were released, our results improved considerably, and it shows that the description of a method is not complete without the details on hyper-parameters. These results are comparable to those in the review, but a final ranking is difficult because of the aforementioned reasons.

## Conclusion

We re-implemented the main method from JAMA 2016; 316(22), but we were not able to get the same performance as reported in that study using publicly available data. The original study had access to data of higher quality than those that are publicly available, and this is likely to account for part of the deviation in results. Gulshan et al showed that the performance levels off around 40 000 (Figure 4A in [8]), and we therefore assume that the reduced size of reproduction data set is not a large source for deviation in the results. The number of grades per image is a possible explanation of Gulshan et al’s superior results, but we cannot quantify the impact. Figure 4B in [8] depicts performance as a function of grades, but there is an overfitting component: 100% vs. 65% specificity for the training and test set, respectively, and it is not possible to distinguish the contribution from the overfitting from that of the low number of grades.

We believe our results show the challenges of reproducing deep learning method results. We therefore recommend the following improvements to the reporting of deep learning methods: (i) use public data or provide detailed data description, (ii) publish source code or all details regarding the pre-processing of the data, and (iii) all hyper-parameters.

The source code of this reproduction study and instructions for running the reproduced experiments are available at https://github.com/mikevoets/jama16-retina-replication.

## Supporting information

S1 Fig Data set distribution. Data set distribution in original study vs. this reproduction study.
(TIF)
