# Supplementary figures and images for: Reproduction study using public data of: Development and validation of a deep learning algorithm for detection of diabetic retinopathy in retinal fundus photographs
Source: PLoS One. 2019 Jun 6;14(6):e0217541. doi: 10.1371/journal.pone.0217541 (PMC6553744; doi:10.1371/journal.pone.0217541)

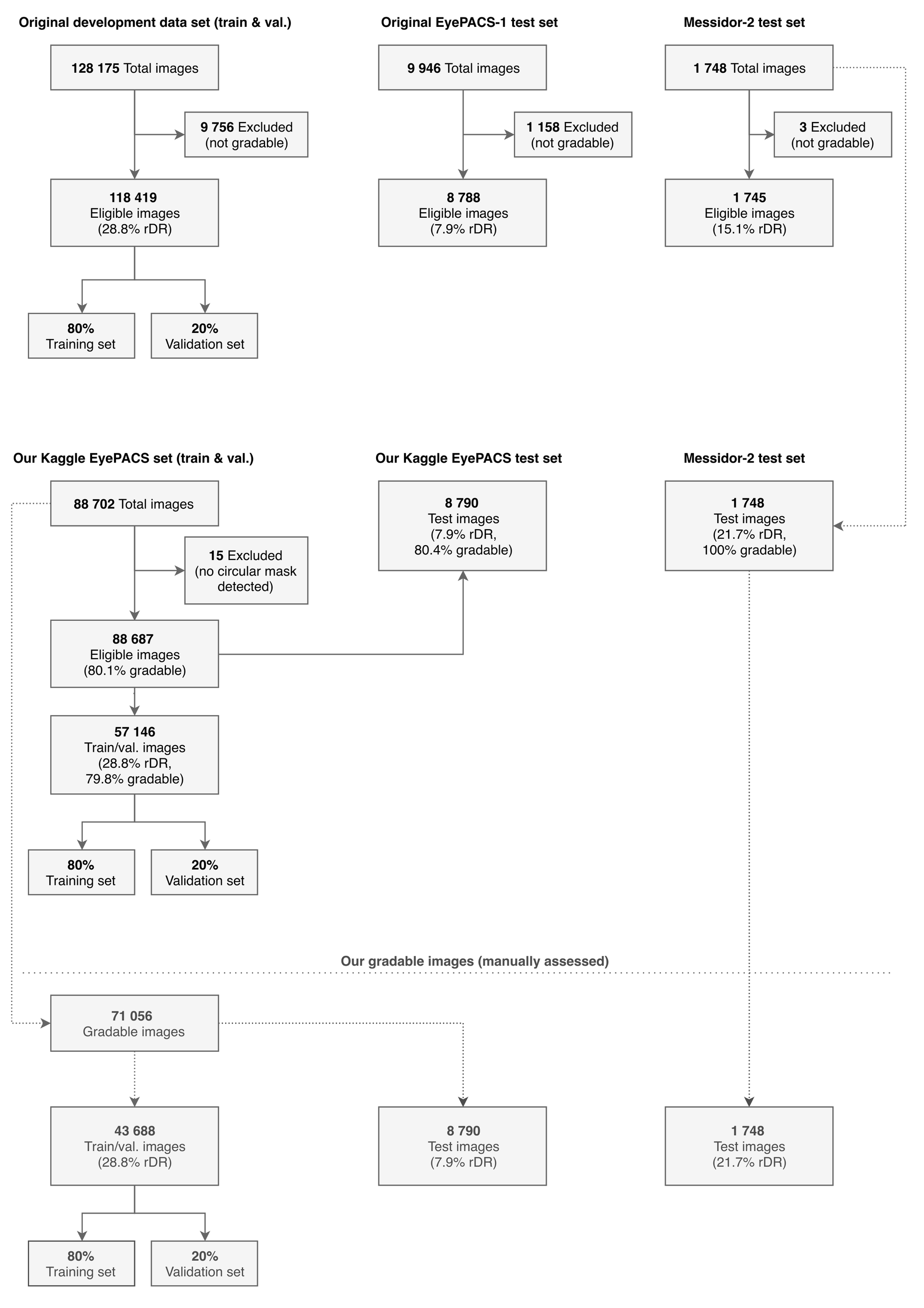

Supplement: S1 Fig — Data set distribution in original study vs. this reproduction study. (TIF) [file pone.0217541.s001.tif]
